# Modelling the emergence of social-bird biological controls to mitigate invasions of the spotted lanternfly and similar invasive pests

**DOI:** 10.1098/rsos.231671

**Published:** 2024-02-21

**Authors:** Daniel Strömbom, Amanda Crocker, Alison Gery, Grace Tulevech, Autumn Sands, Kelly Ward, Swati Pandey

**Affiliations:** Lafayette College, Department of Biology, Easton, PA 18042, USA

**Keywords:** pest management, collective biological control, collective behaviour, social learning

## Abstract

The spotted lanternfly is an emerging global invasive insect pest. Due to a lack of natural enemies where it is invasive, human intervention is required. Extensive management has been applied but the spread continues. Recently, the idea of bird-based biological controls has re-emerged and shown effective in studies. However, it is questionable, if birds are able to effectively control unfamiliar and occasionally toxic invasive pests in short timeframes. Unless, perhaps, the birds are effective social learners and toxicity of the invaders is rare. Here, we introduce a mathematical model for social learning in a great tit-like bird to investigate conditions for the emergence of a collective biological control of a pest that is occasionally toxic, like the lanternfly. We find that the social observation rate relative to the proportion of toxic lanternfly dictate when collective biological controls will emerge. We also implement the social learning model into a model of collective motion in bird-like animals, and find that it produces results consistent with the mathematical model. Our work suggests that social birds may be useful in managing the spotted lanternfly, and that removing the toxicity-inducing preferred host of the lanternfly should be a priority to facilitate this.

## 1. Introduction

The spotted lanternfly (*Lycorma delicatula*) is an insect native to China, India and Vietnam [1] and it is currently considered an invasive pest in South Korea, Japan and the USA [2–4]. Several modelling studies also forecast that it may become invasive on all continents except Antarctica [1,5,6], and it is included on the European and Mediterranean Plant Protection Organization (EPPO) A1 list of quarantine pests that are not yet present in Europe or the Mediterranean [7]. Given that it damages both naturally occurring and economically important flora in countries where it is invasive [8], managing this species where it is already present and limiting its spread is a priority.

Since its confirmed arrival in the USA in 2014, its growth and spread to new areas has been rapid, despite efforts to contain the spread with quarantine zones and to control the populations in infested areas [8,9]. In its native range, egg parasitoid *Anastatus orientalis* [10,11] and nymph parasitoid *Dryinus browni* [12] help control the population. It is believed that a key reason for the lanternfly’s success in the USA and elsewhere where it is invasive is a lack of natural enemies [13]. While a few US organisms have been established to kill lanternfly in the literature, e.g. the wheel bug (*Arilus crisatus*) [13], the stink bug (*Apoecilus cynicus*) [13], entomopathogen *Beauveria bassiana* [14] and egg parasitoid *Ooencyrtus kuvanae* [15], they appear insufficient to control the US lanternfly population.

In lieu of effective natural enemies, human intervention is required and a number of management efforts have been proposed and applied. In particular, quarantines to contain the infestation, and a number of control measures to manage already-infested areas [8]. Control measures currently under consideration, or application, include a variety of insecticides [16], parasite-based biological controls [11,14,17–19], traps [20], egg scraping [21], bark injections and removing the preferred host [22,23]. However, at present, these management efforts appear to be insufficient and there are potential issues with each of them. In particular, traps act only locally, egg scraping may be unfeasible because a large proportion of the eggs are inaccessible [24], a very low percentage of applied insecticides tend to reach the target pest and may have unintended effects on the ecosystem [25,26], and removing the host plants may not be an effective way to limit its food supply given that the lanternfly is known to feed on a wide variety of plants [27,28]. In addition, Strömbom & Pandey [29] have estimated the annual growth rate of the US population to be 5.47 and shown that even for an ideal control that kills 100% of treated lanternfly at least 35% of the lanternfly in all stages must be treated to turn population growth into decline. These proportions are unlikely to be treatable over any large geographical area given that, except for parasite-based biological controls, the proposed control measures require manual application. However, even if distribution issues are ignored, [29] also shows that the proposed controls may have a limited impact on real-world lanternfly populations due to their fast population growth, suggesting that non-standard management approaches might be required; in particular, management approaches that are effective, that do not require manual distribution and that can seek out the lanternfly wherever they may be.

The role of birds for biological control has a long and convoluted history [30]. However, the idea has had a resurgence in recent years [31], partly due to increased interest in sustainable land management [32]. In particular, it has been shown that a number of insectivorous solitary birds are effective biological controls [33–37]. In addition, while their effectiveness has not been investigated, there are numerous anecdotal observations of individual birds eating lanternflies [38]. Finally, Song *et al.* [39] have established that individual oriental tits (*Parus minor*) eat spotted lanternfly that have not fed on the lanternfly’s preferred host, the tree of heaven (*Ailanthus altissima*), but generally avoid eating them if they have fed on this tree. The tree of heaven contains quassinoids that give the lanternfly a number of defences against predators, including making it foul tasting and giving it bright red bands on its wings [39]. Suggesting, perhaps, that if there were no tree of heaven, or other quassinoid producing plants, birds and other predators may begin to consume lanternfly in sufficient quantities to contribute to controlling the population. While this process might be slow with solitary birds like those featuring in [33–37], in group living social birds, like the great tit (*P. major*), this process might be more rapid.

Animals living in social groups often transmit information via cues and signals that enable other animals in the group to benefit from their discoveries or to recruit assistance in completing a task vital to the group [40,41]. For example, ants deposit pheromones to attract nest mates to a food source to help recover it [42], honeybees perform waggle dances to inform hive mates where good food sources are [43], and birds that know how to acquire a particular type of food are observed by uninformed flock mates and the behaviour is copied [44–46]. Fisher and Hinde describe a socially learned feeding behaviour where great tits learn to pierce through the foil cap of milk bottles left outside [44,45]. This behaviour was observed at roughly 30 different sites across the UK, indicating that social learning was a means to acquire novel foraging behaviours. They later suggested that this behaviour was developed independently at each site, with transmission via social learning aiding local adoption of the foraging technique [45]. Similarly, Aplin *et al.* [46] show that information about how to solve a particular problem diffuses through flocks of great tits and that this information stays in the population over multiple generations. In their study, a few wild birds were captured from a forest and taught how to manipulate a puzzle box to gain access to a superior food item. Puzzle boxes were then set up in the forest and the trained birds were released. The information about how to manipulate the puzzle box to access the superior food then spread throughout the population of birds over the coming weeks [46]. A study on black-capped chickadees (*P. atricapillus*) found that these birds were able to replicate the ‘milk bottle opening’ phenomenon by either observing another bird pierce the container lid or by interacting with a previously opened container [47,48]. European starlings (*Sturnus vulgaris*) are also social learners, as seen from a set-up where starlings are taught to obtain food from a box via one of two different methods (pushing or pulling out a plug) and through one of two unique access points (a red versus a black plug) [49]. When these birds were observed by an untrained set of starlings, the observers tended to access the food by removing the same plug via the same method as the bird they watched. This indicates that imitative, observational social learning is also possible in starlings. Whether collective behaviour of this type can be harnessed to combat invasive pests, like the spotted lanternfly, remains unclear both from a theoretical and empirical viewpoint.

Here, we introduce a minimal model of social learning in a great tit-like bird foraging on a lanternfly-like pest population. We derive conditions under which a collective biological control (CBC) will emerge in terms of the model parameters, in particular, the proportion of the pest population that feeds on the toxicity-inducing plant. We study this model both in its non-spatial mathematical form, and when implemented into a spatially explicit model of flocking in bird-like animals [50] that we adapted to include foraging of lanternfly-like agents. Flocking models have been used to explain collective motion in a range of animals across taxa from cells, via fish and birds, to humans [41,51] over the past few decades, but has not yet been used to model social learning in foraging situations of this type. Finally, we outline how our findings may be used in practice and propose experiments to test some of our predictions.

## 2. Models

**Figure 1.**
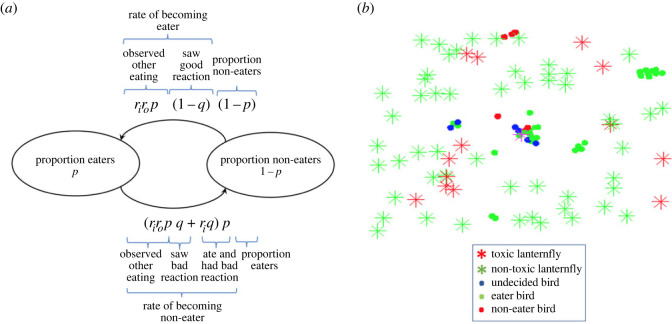
Description of the models. (*a*) Illustration of the mathematical social learning model. There are two compartments, lanternfly eaters and lanternfly non-eaters, and the flow between them is dictated by the process described by each arrow. (*b*) Screenshot of a simulation of the computational model. This has the same heuristic social learning rule programmed into agents, representing the birds, that are moving around in an environment. While moving the agents interact locally with other agents according to a flocking model and with stationary toxic and/or non-toxic lanternfly. Agents also observe interactions between other nearby agent’s interactions with lanternfly, specifically if they had a positive or negative experience eating them and update their state (undecided, eater or non-eater) accordingly.

Consider a population of a great tit bird-like predator and a spotted lanternfly-like prey population. Denote the proportion of the predator population that feed on the prey population by *p* (so a proportion 1 − *p* of the predators do not feed on the prey population), and the proportion of the prey population that feed on the toxicity-inducing host by *q* (so a proportion of 1 − *q* of the prey population do not feed on the toxicity-inducing plant). See figure 1*a*. Predators and prey interact at a rate of *r*_*i*_ (interactions/time step) and each interaction is observed by another predator with probability *r*_*o*_, so the observed interaction rate between predator and prey is *r*_*i*_*r*_*o*_*p*. An observed interaction will be positive with probability 1 − *q* and negative with probability *q*, so the rate of becoming a prey eater (after observing a positive interaction) is *r*_*i*_*r*_*o*_*p*(1 − *q*). Finally, the proportion available to become prey eaters is 1 − *p* so the transition rate from prey non-eaters to prey eaters is *r*_*i*_*r*_*o*_*p*(1 − *q*)(1 − *p*). The transition rate from prey eater to prey non-eater can occur in two ways. Either by having a prey eater observe a negative reaction, which happens at a rate of *r*_*i*_*r*_*o*_*pq*, or if a prey eater has a negative experience itself by eating toxic prey. The latter occurs at a rate of *r*_*i*_*q*, so the total transition rate from prey eater to prey non-eater is (*r*_*i*_*r*_*o*_*pq* + *r*_*i*_*q*)*p*. Thus, the rate of change in the proportion of prey eaters is given by

2.1 $$\frac{dp}{dt}=r_{i}r_{o}p(1-q)(1-p)-(r_{i}r_{o}pq+r_{i}q)p=r_{i}p(r_{o}(1-q-p)-q).$$

### 2.1. Computational model

Here, we model flocking birds that are moving around near their roost site where lanternfly are present. See figure 1*b*. The bird flocking is modelled by a version of the roost site model in [50], and we add social learning and predation of lanternfly upon encounter into this flocking model. At the start of each simulation, all birds are undecided as to whether to try and eat lanternfly, but will try one if encountered with probability *π*, and once it has tried one it will become either a lanternfly eater if it had a good experience (i.e. the lanternfly it ate was not toxic) or a lanternfly non-eater if it had a bad experience (i.e. the lanternfly it ate was toxic). Furthermore, other birds that are within a distance of *R*_*o*_ from a bird that ate a lanternfly will observe whether the experience was good (and then it too becomes a lanternfly eater) or bad (in which case it becomes a non-eater). At the beginning of each simulation, *N*_*l*_ lanternfly are distributed randomly around the roost site and each lanternfly is randomly designated as toxic with probability *q* (model parameter), and non-toxic with probability 1 − *q*. Each simulation proceeds until either all lanternfly present have been consumed, or all birds are lanternfly non-eaters, or the simulation time runs out. Throughout each simulation, we collect the proportion of lanternfly eaters over time, and use this to create plots showing the average proportion of lanternfly eaters over time for different values of *q*, as well as the long-term average proportion of lanternfly eaters as a function *q*. We also parametrize the mathematical model social learning model corresponding to the simulations and present the comparison. Specifically, we estimate the parameters *r*_*o*_ and *r*_*i*_ from the simulations, insert them into the mathematical model for comparison of long-term (and transient) behaviour. See the Methods and calculations section for more details.

## 3. Results

We find that a robust collective biological control emerges in both the mathematical and the computational model. The mathematical model equation (2.1) has the explicit solution

3.1 $$p(t)=\frac{\alpha }{Ke^{-r_{i}\alpha t}+r_{o}},\;\text{with}\;\alpha =r_{o}(1-q)-q\text{ and }K=\frac{\alpha }{p_{0}}-r_{o},$$

and two equilibria

3.2 $$p=0,\;\text{stable when}\;r_{o}<\frac{q}{\left(1-q\right)}$$

and

3.3 $$p=1-\frac{(r_{o}+1)q}{r_{o}},\;\text{stable when}\;r_{o}>\frac{q}{\left(1-q\right)}.$$

The *p* = 0 equilibrium corresponds to a failure of a collective biological control to emerge, i.e. there will be no lanternfly eaters in the system eventually, and this equilibrium is stable when *r*_*o*_ < *q*/(1 − *q*). The *p* = 1 − ((*r*_*o*_ + 1)*q*)/*r*_*o*_ equilibrium represents the collective biological control equilibrium, where this proportion of the birds will be lanternfly eaters eventually, and this equilibrium is stable when *r*_*o*_ > *q*/(1 − *q*). We note that whether or not a collective biological control emerges depend only on the observation rate *r*_*o*_ and the proportion of toxic lanternfly *q*. However, equation (3.1) shows that the rate at which the proportion of eaters approach the equilibrium depends on the interaction rate *r*_*i*_.

**Figure 2.**
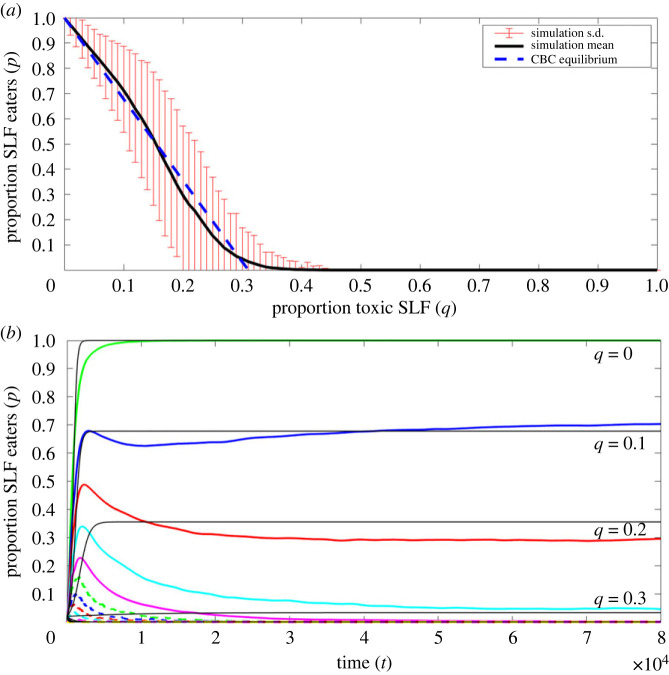
Comparison of the computational and mathematical model results. (*a*) The average long-term proportion of lanternfly eaters as a function of proportion toxic lanternfly (*q*) in simulations (black curve mean with red standard deviation bars) with the corresponding mathematical collective biological control (CBC) equilibrium (3.3) superimposed (dashed blue line). (*b*) The average proportion of lanternfly eaters over time for different proportions of toxic lanternfly (*q*) in simulations (coloured curves) and from the corresponding explicit solution (3.1) (black curves). See electronic supplementary material, figure S1 for an alternative presentation that includes the standard deviations.

Comparison of the mathematical and computational model shows that they produce consistent results (figure 2). In particular, the mathematical prediction of the long-term proportion of eaters (*p*) as a function of proportion of toxic lanternfly (*q*) drops linearly from 1 at *q* = 0 to 0 at *q* ≈ 0.3 which well approximates the average simulation outcome and lies well within the standard deviation bars until *p* reaches 0 (figure 2*a*). We also note that for specific *q* values the long-term behaviour of the mathematical explicit solution formula and the average of the simulations are consistent (figure 2*b*). See electronic supplementary material, figure S1 for a version of figure 2*b* that includes variance information. See the Methods and calculations section for the derivations of the mathematical results and the simulation details.

## 4. Discussion

The spotted lanternfly is an emerging global threat that has proven difficult to deal with where it is invasive [4,9]. Standard management approaches appear insufficient and novel approaches may be required [29]. Animal collectives are capable of solving a wide range of problems [40,41], but have yet to be explored as a possible solution to deal with invasive pests, like the lanternfly.

Our work suggests that collective biological controls may emerge in response to lanternfly infestations if social birds with the right characteristics are present, and that reducing the tree of heaven population may accelerate this process. While the great tit (*P. major*) itself will be a viable candidate if/when the lanternfly arrives in Europe [7], and the closely related oriental tit (*P. minor*) is a viable candidate in South Korea at present, they are not a viable options for the current US infestation because they do not exist in the USA. Fortunately, there are several social birds currently present in the USA that may be suitable candidates. In particular, highly social birds including chickadees (a relative of the great tit) and starlings have been anecdotally observed to eat lanternfly [38]. If there were no bad side-effects to eating the lanternfly, perhaps this behaviour would spread socially and chickadee or starling collective biological controls emerge. However, our work suggests that for this to occur locally the tree of heaven, and/or other plants containing quassinoids, must be effectively removed from the area. Figure 2*a* shows that the equilibrium proportion of lanternfly eaters drops quickly with proportion of toxic lanternfly, meaning that if even a low number of toxicity-inducing host trees are present the process may fail. In addition to removing tree of heaven to facilitate a spontaneous natural process, perhaps it could be facilitated further by training birds to eat the lanternfly in tree of heaven-free areas, similar to training the birds to open puzzle boxes in [46]. The milk bottle phenomenon observed in great tits [44,45] and blue tits (*Cyanistes caeruleus*) [52] demonstrates the ability of social birds to engage in observational learning in their typical habitat and in artificial testing environments. Starlings also exhibit imitative learning [49], and both chickadees [53] and warblers (*Dendroica virens* and *D. caerulescen*) [54] have been observed to adopt learning behaviours associated with foraging. Additionally, chickadees in the presence of conspecifics have an increased likelihood to open cream containers, even if they have not previously observed the behaviour [47,48]. The presence of fellow chickadees may reduce fear [55] or elicit foraging behaviours that promote exploratory food discovery. This could be beneficial, especially in regions where the lanternfly is newly present, as birds may be more willing to feed on the foreign insect when in groups. How social birds like chickadees and starlings are also varies with the season [56–58] so the timing of tree of heaven removal and other social learning promoting activities may be worth considering before deployment. However, because the lanternfly is present in the nymphs and adult stages from May–December and in the adult stage from July–December [8] there should be ample time outside the less social breeding season for social learning to occur. Experimental work to test the merit of the basic idea proposed here should be possible given that the experiments in [39] have already been carried out, and there are labs in the quarantine zone in the USA that have access to both suitable social birds (starlings and chickadees) and to lanternfly. As a first viability test, it should be determined if a flock of captive social birds spontaneously, or by training a few individuals, will learn to eat toxin-free lanternfly. The same experiment could be used to assess the efficacy of the bird biological control by measuring the proportion of available lanternfly that were killed during the experiment(s) (if any) and the results directly compared with previous studies of mid-winter chipping [21], egg parasite (*O. kuvanae*) [59] entomopathogen (*B. bassiana*) [14], insecticide Bifenthrin [16], insecticide Chlorpyrifos [16,60] and others [9].

Here, we provide a complete analysis of the mathematical social learning model that shows that a collective biological control does emerge under a range of conditions. However, more data are required in each specific situation to make robust predictions. In particular, *r*_*o*_ and *r*_*i*_ must be estimated for each bird community and environment, but from a management perspective these parameters probably cannot be affected anyway, the readily affectable parameter is *q*. Once *r*_*o*_ and *r*_*i*_ have been estimated for a given situation, then the *q* threshold for emergence of a lanternfly eating collective can be calculated and set as an upper-level target for local eradication. For *q* above this threshold, collective biological controls will not emerge and contribute to management, so focus should be on reducing *q* via removal of the tree of heaven. Fortunately the removal of the tree of heaven has been a prioritized control effort over the past few years [9], suggesting that regions in which collective biological controls of the type described here might emerge may be increasing or already exist locally. In fact, some indication of their existence may be present in the data already collected for the work in [38]. Specifically, if several different individual starlings, chickadees or other social birds have been observed eating lanternfly around the same region this might be an indicator, and experimental studies in addition to observational studies could be launched in such regions to determine if a bird collective biological control is operating there.

A bird biological control could have several benefits over currently used control measures. In particular, most currently used controls require more or less manual distribution and application, for example, insecticides [16], traps [20] and egg scraping [21], and are therefore unlikely to be effective over larger geographical areas. To remedy this issue, biological controls, where other organisms, native or imported, are used to control the pest, have been proposed and studied; in particular, parasite-based biological controls [14,17–19]. Unfortunately, they may also be ineffective in the wild due to their low effectiveness [29]. A bird biological control overcomes some of the issues associated with current controls. In particular, social birds can go wherever the lanternfly is present without manual distribution, they are an environmentally better option than insecticides [32], and their efficacy could potentially be substantial if they start consuming lanternfly collectively. Given that even solitary birds are effective biological controls [33–37], insectivorous birds are key controllers of insects globally [61], and animal collectives often are supremely effective foragers [40,41]. Perhaps they can even help mediate a particularly persistent issue with conventional controls, namely that the last few pest organisms are the most difficult to find and eliminate. Not only are birds moving and exploring animals that regularly hunt in the region where the lanternflies live, but they can also reach the large number of lanternflies that are present high up in trees (above 6 m) [24] and on other structures where conventional controls are difficult to deploy. However, the actual potential real-world efficacy of social birds as controls of the spotted lanternfly and how they might be integrated into existing integrated pest management programmes will ultimately have to be assessed by the US Department of Agriculture (USDA) and the departments of agriculture in affected states, because they are the only actors that may have access to the information, expertise, resources and a holistic enough view of current management protocols that are required for this.

At present, it seems that managing the spotted lanternfly in the USA, and elsewhere, is a losing battle. Despite significant efforts on many fronts, by many actors, and in many ways its growth and spread continues. We believe that exploring and adding more unconventional components to the conventional management approaches currently used may increase our chances of more effectively managing the lanternfly. In particular, given their extraordinary effectiveness in other contexts, we propose effort goes into exploring the potential of animal collectives to be part of integrated pest management programmes. Here, we have illustrated one aspect of this idea via social birds as a first step in this direction.

## 5. Methods and calculations

### 5.1. Calculating the equilibria and their stability

We employ standard qualitative analysis for autonomous differential equations [62] to find the equilibria and determine their stability properties (equations (3.2) and (3.3)). This involves finding the equilibria y* of the equation *y*′ = *f*(*y*) by solving *f*(y*) = 0, and then for each equilibrium y* determine its stability properties via the criteria that y* is stable if *f*′(y*) < 0.

#### 5.1.1. Equilibria

Our model equation (2.1) is autonomous and we find the equilibria by solving *r*_*i*_*p*(*r*_*o*_(1 − *q* − *p*) − *q*) = 0 for *p*. This equation is satisfied when *p* = 0 (Equilibrium 1) or (*r*_*o*_(1 − *q* − *p*) − *q*) = 0, and solving the latter equation for *p* gives *p* = 1 − (*r*_*o*_ + 1)*q*)/*r*_*o*_ (Equilibrium 2).

#### 5.1.2. Stability

The derivative of the function *f*(*p*) = *r*_*i*_*p*(*r*_*o*_(1 − *q* − *p*) − *q*) with respect to *p* is *f*′(*p*) = *r*_*i*_(*r*_*o*_(1 − *q* − 2*p*) − *q*) and we substitute each equilibrium into this expression and solve the resulting inequality *f*′(*p*) < 0 for *p* to find the stability properties of each equilibrium. Throughout we assume that *r*_*i*_, *r*_*o*_ > 0 and *q* ∈ (0, 1).

**Equilibrium 1 (*p* = 0)**

$$\begin{array}{rl} & f^{\prime }(0)=r_{i}(r_{o}(1-q)-q),\\ & r_{i}(r_{o}(1-q)-q)<0,\\ & r_{o}(1-q)<q\\ \;\mathrm{and}\; & r_{o}<\frac{q}{\left(1-q\right)}. \end{array}$$

So the equilibrium *p* = 0 is stable when *r*_*o*_ < *q*/(1 − *q*).

**Equilibrium 2 (*p* = 1 − (*r*_*o*_ + 1)*q*)/*r*_*o*_)**

$$\begin{array}{rl} & f^{\prime }((1-(r_{o}+1)q)/r_{o})=r_{i}(r_{o}(q-1)+q),\\ & r_{i}(r_{o}(q-1)+q)<0,\\ & r_{o}(q-1)<-q,\\ & r_{o}>-q/(q-1)\\ \;\mathrm{and}\; & r_{o}>q/(1-q). \end{array}$$

So the equilibrium *p* = 1 − (*r*_*o*_ + 1)*q*)/*r*_*o*_ is stable when *r*_*o*_ > *q*/(1 − *q*).

### 5.2. Finding the explicit solution

The model equation (2.1) is separable and straightforward to solve as follows:

$$\begin{array}{rl} \frac{dp}{dt} & =r_{i}p(r_{o}(1-q-p)-q),\\ & \int \frac{1}{p(r_{o}(1-q-p)-q)}\;dp=\int r_{i}\;dt,\\ & \frac{\ln\left(r_{o}(q+p-1)+q\right)-\ln\;p}{r_{o}(q-1)+q}=r_{i}t+K,\\ & \frac{r_{o}(q+p-1)+q}{p}=Ke^{r_{i}(r_{o}(1-q)-q)t}\\ \;\mathrm{and}\; & p(t)=\frac{r_{o}(1-q)-q}{r_{o}-Ke^{-r_{i}(r_{o}(1-q)-q)t}}. \end{array}$$

The general solution is therefore

$$p(t)=\frac{\alpha }{r_{o}-Ke^{-r_{i}\alpha t}},$$

with *α* = *r*_*o*_(1 − *q*) − *q*.

If we denote the initial proportion of lanternfly eaters by *p*_0_, then the constant *K* is determined by

$$p(0)=\frac{\alpha }{r_{o}-Ke^{-r_{i}\alpha 0}}=\frac{\alpha }{r_{o}-K}=p_{0},$$

so *K* = *r*_*o*_ − *α*/*p*_0_ and the particular solution will then be

$$p(t)=\frac{\alpha }{r_{o}+(\alpha /p_{0}-r_{o})e^{-r_{i}\alpha t}}.$$

### 5.3. Simulations of the computational model and comparison with the mathematical model

For the underlying flocking model itself [50], we use the same interactions and parameter values as for their roosting site model. See eqn (3) and §2.2 in [50]. The additional parameters that are introduced in our version is the probability that an undecided bird spontaneously tries to eat a lanternfly which we set to *π* = 1/100 to ensure that once the process has started the social learning rate is substantially higher than the spontaneous learning rate. The range over which other birds observe other bird-lanternfly interactions is set to *R*_*o*_ = 6, equal to the bird–bird interaction radius *R* in [50]. The number of birds is set to *N* = 50. At the start of each simulation, *N*_*l*_ = 500 lanternfly are distributed randomly in a region of size 150 × 150 units of area centred at the roosting site. Each lanternfly is randomly assigned to be toxic with probability *q*, and non-toxic with probability 1 − *q*. *q* is the main parameter of interest and we explore its full range [0, 1].

To determine how the long-term proportion of lanternfly eaters *p* varies with proportion of toxic lanternfly *q* we ran 2000 simulations for each value of *q* from 0 to 1 in increments of 0.01 for 6 × 10^6^ time steps. Unless the simulation terminates early because all lanternfly have been eaten or all birds have become non-eaters. Then, for each *q*, we calculated the mean and standard deviation of the recorded final value in each of the 2000 simulations, and this information was used to create the simulation parts of figure 2*a*. Through simulations we also estimate the bird–lanternfly interaction rate (*r*_*i*_ = 0.01) and the proportion of such interactions that are observed by other birds (*r*_*o*_ = 0.45) by counting the number of such interactions and whether they were observed by other birds. The *r*_*o*_ = 0.45 value was then substituted in equation (3.2) and plotted as a function of *q* to create the mathematical part (CBC equilibrium) of figure 2*a*.

To determine how the proportion of lanternfly eaters vary over time for different proportions of toxic lanternfly *q*, we ran 5000 simulations for each value of *q* from 0 to 1 in increments of 0.1 for 6 × 10^6^ time steps, and collected the proportion of lanternfly eaters in each time step in each simulation. Then, for each *q*, we calculated the mean and standard deviation of *p* over the 5000 simulations at each time step. A rolling average of the mean *p* over time for each *q* was then used to create the computational parts of figure 2*b*. Electronic supplementary material, figure S1 shows the raw means and standard deviations of the data used to create figure 2*b*. The simulation estimated *r*_*o*_ = 0.45 and *r*_*i*_ = 0.01 values were then substituted in equation (3.1) and plotted as a function of *q* to create the mathematical parts (explicit solutions for *q* = 0, 0.1, …, 0.9, 1) of figure 2*b*.

See the Data accessibility statement for how to access the code required to replicate the computational results presented in this manuscript.

## Data Availability

Data and relevant code for this research work are stored in GitHub: https://github.com/danielstrombom/Facilitating and have been archived within the Zenodo repository: https://doi.org/10.5281/zenodo.10472351 [63]. Supplementary material is available online [64].

## References

[1] Jung JM, Jung S, Byeon D, Lee WH. 2017 Model-based prediction of potential distribution of the invasive insect pest, spotted lanternfly *Lycorma delicatula* (Hemiptera: Fulgoridae), by using CLIMEX. J. Asia-Pac. Biodivers. **10**, 532-538. (10.1016/j.japb.2017.07.001)

[2] Dara SK, Barringer L, Arthurs SP. 2015 *Lycorma delicatula* (Hemiptera: Fulgoridae): a new invasive pest in the United States. J. Integr. Pest Manage. **6**, 20. (10.1093/jipm/pmv021)

[3] Nakashita A, Wang Y, Lu S, Shimada K, Tsuchida T. 2022 Ecology and genetic structure of the invasive spotted lanternfly *Lycorma delicatula* in Japan where its distribution is slowly expanding. Sci. Rep. **12**, 1543. (10.1038/s41598-022-05541-z) 35105894 PMC8807778

[4] Zhang Y, Bao K, Xin B, Cao L, Wei K, Dang Y, Yang Z, Lv Z, Wang X. 2023 The biology and management of the invasive pest spotted lanternfly, *Lycorma delicatula* White (Hemiptera: Fulgoridae). J. Plant Dis. Protect. 130, 1155-1174. (10.1007/s41348-023-00794-w)

[5] Wakie TT, Neven LG, Yee WL, Lu Z. 2020 The establishment risk of *Lycorma delicatula* (Hemiptera: Fulgoridae) in the United States and globally. J. Econ. Entomol. **113**, 306-314. (10.1093/jee/toz259) 31579914

[6] Namgung H, Kim MJ, Baek S, Lee JH, Kim H. 2020 Predicting potential current distribution of *Lycorma delicatula* (Hemiptera: Fulgoridae) using MaxEnt model in South Korea. J. Asia-Pac. Entomol. **23**, 291-297. (10.1016/j.aspen.2020.01.009)

[7] European and Mediterranean Plant Protection Organization. 2023. EPPO A1 List of pests recommended for regulation as quarantine pests. See https://www.eppo.int/ACTIVITIES/plant_quarantine/A1_list (accessed 22 August 2023).

[8] Urban JM. 2020 Perspective: shedding light on spotted lanternfly impacts in the USA. Pest Manag. Sci. **76**, 10-17. (10.1002/ps.5619) 31525270

[9] Urban JM, Leach H. 2023 Biology and management of the spotted lanternfly, *Lycorma delicatula* (Hemiptera: Fulgoridae), in the United States. Annu. Rev. Entomol. **68**, 151-167. (10.1146/annurev-ento-120220-111140) 36206772

[10] Kim JG, Lee EH, Seo YM, Kim NY. 2011 Cyclic behavior of *Lycorma delicatula* (Insecta: Hemiptera: Fulgoridae) on host plants. J. Insect Behav. **24**, 423. (10.1007/s10905-011-9266-8)

[11] Wu Y et al. 2023 Cryptic genetic diversity and associated ecological differences of *Anastatus orientalis*, an egg parasitoid of the spotted lanternfly. Front. Insect Sci. **3**, 1154651. (10.3389/finsc.2023.1154651) PMC10926478 38469524

[12] Yan J, Yu X, Qin X, Wang F, Bo L. 2008 Study on the biology of *Dryinus browni*. Shandong Forest. Sci. Technol. **2008**, 16-18.

[13] Barringer LE, Smyers E. 2016 Predation of the spotted lanternfly, *Lycorma delicatula* (White) (Hemiptera: Fulgoridae) by two native Hemiptera. Entomol. News **126**, 71-73. (10.3157/021.126.0109)

[14] Clifton EH, Hajek AE, Jenkins NE, Roush RT, Rost JP, Biddinger DJ. 2020 Applications of *Beauveria bassiana* (Hypocreales: Cordycipitaceae) to control populations of spotted lanternfly (Hemiptera: Fulgoridae), in semi-natural landscapes and on grapevines. Environ. Entomol. **49**, 854-864. (10.1093/ee/nvaa064) 32488261

[15] Liu H. 2019 Oviposition substrate selection, egg mass characteristics, host preference, and life history of the spotted lanternfly (Hemiptera: Fulgoridae) in North America. Environ. Entomol. **48**, 1452-1468. (10.1093/ee/nvz099) 31651025

[16] Leach H, Biddinger DJ, Krawczyk G, Smyers E, Urban JM. 2019 Evaluation of insecticides for control of the spotted lanternfly, *Lycorma delicatula*, (Hemiptera: Fulgoridae), a new pest of fruit in the Northeastern US. Crop Protect. **124**, 104833. (10.1016/j.cropro.2019.05.027)

[17] Choi MY, Yang ZQ, Wang XY, Tang YL, Hou ZR, Kim JH, Byeon YW. 2014 Parasitism rate of egg parasitoid *Anastatus orientalis* (Hymenoptera: Eupelmidae) on *Lycorma delicatula* (Hemiptera: Fulgoridae) in China. Korean J. Appl. Entomol. **53**, 135-139. (10.5656/KSAE.2014.01.1.075)

[18] Yang ZQ, Choi WY, Cao LM, Wang XY, Hou ZR. 2015 A new species of *Anastatus* (Hymenoptera: Eulpelmidae) from China, parasitizing eggs of *Lycorma delicatula* (Homoptera: Fulgoridae). Zool. Syst. **40**, 290-302.

[19] Lee DH, Park YL, Leskey TC. 2019 A review of biology and management of *Lycorma delicatula* (Hemiptera: Fulgoridae), an emerging global invasive species. J. Asia-Pac. Entomol. **22**, 589-596. (10.1016/j.aspen.2019.03.004)

[20] Francese JA, Cooperband MF, Murman KM, Cannon SL, Booth EG, Devine SM, Wallace MS. 2020 Developing traps for the spotted lanternfly, *Lycorma delicatula* (Hemiptera: Fulgoridae). Environ. Entomol. **49**, 269-276. (10.1093/ee/nvz166) 31990325

[21] Cooperband MF, Mack R, Spichiger SE. 2018 Chipping to destroy egg masses of the spotted lanternfly, *Lycorma delicatula* (Hemiptera: Fulgoridae). J. Insect Sci. **18**, 7. (10.1093/jisesa/iey049) PMC6007429 29868780

[22] Rowe LM, Higman PJ, Enander HD. 2020 *Screening the Michigan Forest Inventory and Midwest Invasive Species Network databases to locate host plants of Lycorma delicatula (spotted lanternfly) in Michigan*. Technical report.

[23] APHIS 2018. *Spotted lanternfly control program in the mid-Atlantic region–environmental assessment*. Washington, DC: USDA APHIS.

[24] Keller J, Rost J, Hoover K, Urban J, Leach H, Porras M, Walsh B, Bosold M, Calvin D. 2020 Dispersion patterns and sample size estimates for egg masses of spotted lanternfly (Hemiptera: Fulgoridae). Environ. Entomol. **49**, 1462-1472. (10.1093/ee/nvaa107) 33315076

[25] Pimentel D, Levitan L. 1986 Pesticides: amounts applied and amounts reaching pests. Bioscience **36**, 86-91. (10.2307/1310108)

[26] Dent D, Binks RH. 2020 Insect pest management. Wallingford, UK: CABI.

[27] Murman K et al. 2020 Distribution, survival, and development of spotted lanternfly on host plants found in North America. Environ. Entomol. **49**, 1270-1281. (10.1093/ee/nvaa126) 33128562

[28] Barringer L, Ciafré CM. 2020 Worldwide feeding host plants of spotted lanternfly, with significant additions from North America. Environ. Entomol. **49**, 999-1011. (10.1093/ee/nvaa093) 32797186

[29] Strömbom D, Pandey S. 2021 Modeling the life cycle of the spotted lanternfly (*Lycorma delicatula*) with management implications. Math. Biosci. **340**, 108670. (10.1016/j.mbs.2021.108670) 34302819

[30] Evenden MD. 1995 The laborers of nature: economic ornithology and the role of birds as agents of biological pest control in North American agriculture, ca. 1880–1930. Forest Conserv. Hist. **39**, 172-183. (10.2307/3983958)

[31] Garcia K, Olimpi EM, Karp DS, Gonthier DJ. 2020 The good, the bad, and the risky: can birds be incorporated as biological control agents into integrated pest management programs? J. Integr. Pest Manage. **11**, 11. (10.1093/jipm/pmaa009)

[32] Foley JA et al. 2005 Global consequences of land use. Science **309**, 570-574. (10.1126/science.1111772) 16040698

[33] Marquis RJ, Whelan CJ. 1994 Insectivorous birds increase growth of white oak through consumption of leaf-chewing insects. Ecology **75**, 2007-2014. (10.2307/1941605)

[34] Karp DS, Mendenhall CD, Sandí RF, Chaumont N, Ehrlich PR, Hadly EA, Daily GC. 2013 Forest bolsters bird abundance, pest control and coffee yield. Ecol. Lett. **16**, 1339-1347. (10.1111/ele.12173) 23981013

[35] Maas B, Clough Y, Tscharntke T. 2013 Bats and birds increase crop yield in tropical agroforestry landscapes. Ecol. Lett. **16**, 1480-1487. (10.1111/ele.12194) 24131776

[36] Maas B et al. 2016 Bird and bat predation services in tropical forests and agroforestry landscapes. Biol. Rev. **91**, 1081-1101. (10.1111/brv.12211) 26202483

[37] Heath SK, Long RF. 2019 Multiscale habitat mediates pest reduction by birds in an intensive agricultural region. Ecosphere **10**, e02884. (10.1002/ecs2.2884)

[38] Johnson AE, Cornell A, Hermann S, Zhu F, Hoover K. 2023 Using community science to identify predators of spotted lanternfly, *Lycorma delicatula* (Hemiptera: Fulgoridae), in North America. Bull. Entomol. Res. 113, 637-644. (10.1017/S0007485323000317) 37614127

[39] Song S, Kim S, Kwon SW, Lee SI, Jablonski PG. 2018 Defense sequestration associated with narrowing of diet and ontogenetic change to aposematic colours in the spotted lanternfly. Sci. Rep. **8**, 16831. (10.1038/s41598-018-34946-y) 30442911 PMC6237927

[40] Sumpter DJ. 2010 Collective animal behavior. Princeton, NJ: Princeton University Press.

[41] Ward A, Webster M. 2016 Sociality: the behaviour of group-living animals, vol. 407. New York, NY: Springer.

[42] Hölldobler B, Wilson EO. 1990 The ants. Cambridge, MA: Harvard University Press.

[43] Biesmeijer JC, Seeley TD. 2005 The use of waggle dance information by honey bees throughout their foraging careers. Behav. Ecol. Sociobiol. **59**, 133-142. (10.1007/s00265-005-0019-6)

[44] Fisher J. 1949 The opening of milkbottles by birds. Brit. Birds **42**, 347-357.

[45] Hinde R, Fisher J. 1972 Some comments on the re-publication of two papers on the opening of milk bottles by birds. In Function and evolution of behavior (eds PH Klopfer, JP Hailman), pp. 377-378. Reading, MA: Addison-Wesley.

[46] Aplin LM, Farine DR, Morand-Ferron J, Cockburn A, Thornton A, Sheldon BC. 2015 Experimentally induced innovations lead to persistent culture via conformity in wild birds. Nature **518**, 538-541. (10.1038/nature13998) 25470065 PMC4344839

[47] Sherry D, Galef B. 1984 Cultural transmission without imitation: milk bottle opening by birds. Anim. Behav. **32**, 937-938. (10.1016/S0003-3472(84)80185-2)

[48] Sherry D, Galef B. 1990 Social learning without imitation: more about milk bottle opening by birds. Anim. Behav. **40**, 987-989. (10.1016/S0003-3472(05)81004-8)

[49] Campbell F, Heyes C, Goldsmith A. 1999 Stimulus learning and response learning by observation in the European starling, in a two-object/two-action test. Anim. Behav. **58**, 151-158. (10.1006/anbe.1999.1121) 10413551

[50] Strömbom D, Nickerson S, Futterman C, DiFazio A, Costello C, Tunstrøm K. 2022 Bistability and switching behavior in moving animal groups. Northeast J. Complex Syst. (NEJCS) **4**, 1. (10.22191/nejcs/vol4/iss1/1)

[51] Vicsek T, Zafeiris A. 2012 Collective motion. Phys. Rep. **517**, 71-140. (10.1016/j.physrep.2012.03.004)

[52] Aplin L, Sheldon B, Morand-Ferron J. 2013 Milk bottles revisited: social learning and individual variation in the blue tit, *Cyanistes caeruleus*. Anim. Behav. **85**, 1225-1232. (10.1016/j.anbehav.2013.03.009)

[53] Heinrich B, Collins S. 1983 Caterpillar leaf damage, and the game of hide-and-seek with birds. Ecology **64**, 592-602. (10.2307/1939978)

[54] Whelan C. 1989 An experimental test of prey distribution learning in two paruline warblers. Condor **91**, 113-119. (10.2307/1368154)

[55] Clayton D. 1978 Socially facilitated behavior. Q. Rev. Biol. **53**, 373-392. (10.1086/410789)

[56] Kessel B. 1957 A study of the breeding biology of the European starling (*Sturnus vulgaris* L.) in North America. Am. Midland Naturalist 58, 257-331. (10.2307/2422615)

[57] Tinbergen JM. 1981 Foraging decisions in starlings (*Sturnus vulgaris* L). *Ardea* **69**, 1–67. (10.5253/arde.v69.p1)

[58] Kluyver H. 1961 Food consumption in relation to habitat in breeding chickadees. Auk 78, 532-550. (10.2307/4082187)

[59] Liu H. 2019 Occurrence, seasonal abundance, and superparasitism of *Ooencyrtus kuvanae* (Hymenoptera: Encyrtidae) as an egg parasitoid of the spotted lanternfly (*Lycorma delicatula*) in North America. Forests **10**, 79. (10.3390/f10020079)

[60] Shin YH, Moon SR, Yoon CM, Ahn KS, Kim GH. 2010 Insecticidal activity of 26 insectcides against eggs and nymphs of *Lycorma delicatula* (Hemiptera: Fulgoridae). Korean J. Pesticide Sci. **14**, 157-163.

[61] Nyffeler M, Şekercioğlu ÇH, Whelan CJ. 2018 Insectivorous birds consume an estimated 400–500 million tons of prey annually. Sci. Nat. **105**, 1-13. (10.1007/s00114-018-1571-z) PMC6061143 29987431

[62] Otto SP, Day T. 2007 A biologist’s guide to mathematical modeling in ecology and evolution. Princeton, NJ: Princeton University Press.

[63] Strömbom D, Gery A. 2024 danielstrombom/Facilitating: Facilitating. Zenodo. (10.5281/zenodo.10472351)

[64] Strömbom D, Crocker A, Gery A, Tulevech G, Sands A, Ward K, Pandey S. 2024 Modelling the emergence of social-bird biological controls to mitigate invasions of the spotted lanternfly and similar invasive pests. *Figshare*. (10.6084/m9.figshare.c.7073632) PMC10878819 38384778

